# A Rare Case of Abdominal Tumor: Mesenteric Cyst

**DOI:** 10.7759/cureus.29949

**Published:** 2022-10-05

**Authors:** Yitong Xiao, Sanket Chaudhari, Tauqir Khattak, Frederick Tiesenga

**Affiliations:** 1 Surgery, Saint James School of Medicine, Park Ridge, USA; 2 School of Medicine, Saint James School of Medicine, Park Ridge, USA; 3 Internal Medicine, Saint James School of Medicine, Park Ridge, USA; 4 General Surgery, West Suburban Medical Center, Oak Park, USA

**Keywords:** constipation, atypical abdominal pain, abdominal cyst, tumor surgery, primary mesenteric cyst

## Abstract

Mesenteric cysts are rare, usually benign, tumors that typically present asymptomatically and are found incidentally during evaluation for nonspecific abdominal symptoms. We present the case of a 41-year-old African American female who was found to have a mesenteric cyst in her jejunum during the evaluation of abdominal pain, nausea, and constipation that she had been experiencing for six weeks. Pre-operatively, an abdominal CT scan showed a 6x4x6 cm mesenteric cystic lesion in the right mid-abdomen, which was then successfully resected off the mesentery of the jejunum laparoscopically. Her postoperative course was uneventful and she was discharged home without complication a few hours after her procedure with appropriate follow-up.

## Introduction

Mesenteric cysts are rare, benign, intra-abdominal tumors with an incidence of one case per 250,000 hospital admissions. They are typically asymptomatic but can have variable presentations depending on location and morphology. Therefore, they are typically found incidentally during abdominal imaging or laparotomy when investigating non-specific abdominal symptoms [1]. Cysts can occur anywhere in the mesentery from the duodenum to the sigmoid. A review of 162 patient cases showed 60% of cysts occurring in the small bowel mesentery, 24% in the large bowel mesentery, and 14.5% in the retroperitoneum [2]. They can present as simple or multiple cysts, unilocular or multilocular, and contain serous, purulent, chylous, or hemorrhagic fluid. Multiple theories have been proposed as to the etiology of these lesions such as trauma, infection, and insufficient lymphatic flow; without any sufficient evidence supporting one over the other. Although malignancy is quite rare, occurring in only 3% of cases, resection of the cyst is the treatment of choice due to the low rate of recurrence and excellent prognosis [3].

## Case presentation

A 41-year-old African American female presented to the emergency room with abdominal pain, nausea, and constipation for six weeks. Her abdominal pain was constant without precipitating or alleviating factors. The patient reported that her abdominal pain was moderate and originated at the right lower quadrant, and it started progressing and shooting up to her right shoulder in the past few days. The patient also reported that she was having constipation in the past few weeks, and she has tried ingesting charcoal to alleviate her symptoms. According to the patient, charcoal was helpful with her constipation and nausea, but the abdominal pain was unbearable. The patient denied any blood in her bowel movement, fevers, and sick contacts. 

**Figure 1:**
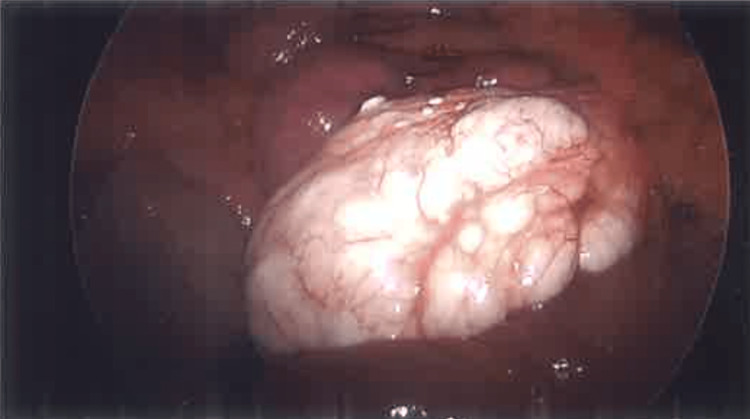
Image taken by laparoscopic camera showing multiloculated cyst wrapped around the jejunum, with an approximate measurement of 6 x 4 x 6cm. It spared the anterior one-third of the affected jejunum.

**Figure 2:**
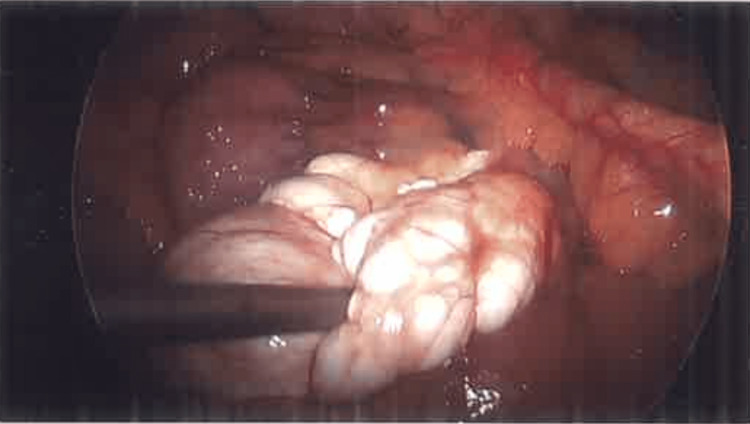
Image taken by laparoscopic camera showing posterior attachment of the mesenteric cyst to the jejunum.

The patient had a cesarean section in 2007, a laparoscopic gastric band procedure in 2010, an abdominoplasty procedure in 2018 for cosmetic purposes, and a preauricular cyst removal procedure in 2021. Due to leakage from the gastric band, she was scheduled at our outpatient surgery clinic for a laparoscopic gastric band revision procedure in three weeks. Beyond her past surgical history, the patient’s medical history was unremarkable. She was not taking any type of home medications. Based on the patient's clinical presentation and surgical history, an imaging study was suggested at the emergency department and the computerized tomography (CT) scan indicated a 6x4x6 cm mesenteric cystic lesion in the right mid abdomen (Figures 1, 2).

After a thorough discussion with the surgeon about her clinical symptoms and imaging report, a surgical treatment plan was proposed for her lap band revision and mesenteric cyst removal. The patient was brought to the operating room and after sedation was provided, placed into a supine position. The area of the surgical incision was then prepped and draped. Timeout was performed. She received appropriate IV antibiotics within one hour of skin incision. An incision over the mass (mesenteric cysts) was then made with a scalpel. Subcutaneous tissue was divided with electrocautery, and the mass was discovered, excised, and sent to pathology. There was no gross involvement of the fascia. The wound was washed out. Hemostasis was achieved. Wounds were closed in layers anatomically. The patient tolerated the procedure well and was awoken and taken to the recovery room in stable condition. The patient was discharged from our hospital a few hours after the procedure without any postoperative complications. 

## Discussion

A mesenteric cyst is one of the rarest abdominal tumors. The incidence of this tumor is around one in 100,000-250,000 [4]. Mesenteric cysts can be found anywhere in the abdominal mesentery. In a study done by Ravano et al., the most common location of mesenteric cysts was the small bowel mesentery, seen in about 60% of all cases. A mesenteric cyst can occur at any age; however, it seems more common in patients younger than 15 years old [5].

The origin of mesenteric cysts is still uncertain [6]. Most of the time, mesenteric cysts are asymptomatic; however, in some patients, they can cause symptoms or other complications. Symptoms can include pain, nausea, vomiting, constipation, and diarrhea. Mesenteric cysts can grow to extreme sizes, to the point of causing small bowel obstruction, abdominal deformation, or resembling ascites [5]. In rare cases, they can even perforate. 

The diagnosis of a mesenteric cyst is usually done by imaging techniques such as CT scans, ultrasound, and MRI. For example, the CT scan will show a cystic lesion, and depending on the content of the cyst it can show anywhere from hypodense to dense lesions on the scan. Due to the rarity of this lesion, a mesenteric cyst can be misdiagnosed on imaging depending on its location. Common misdiagnoses include pancreatic pseudocysts and ovarian cysts. A case report by Theodoridis et al. describes a case where the initial diagnosis was presumed to be a para-ovarian cyst on ultrasound [7]. It was not until the laparoscopic procedure that the simple cyst was seen, diagnosed, and excised. 

Treatment for the cyst differs based on the symptoms. For an asymptomatic cyst, no treatment needs to be done. For a cyst that’s causing symptoms, the treatments include anywhere from drainage, aspiration, or excision. The definitive treatment is excision of the cyst via laparoscopic surgery as other methods have shown higher rates of recurrence [7]. Laparoscopic surgical removal was the treatment of choice in our case, mainly to prevent some secondary complications associated with mesenteric cysts including volvulus, spillage of infective fluid, herniation of the bowel into an abdominal defect, and obstruction [8]. 

**Figure 3:**
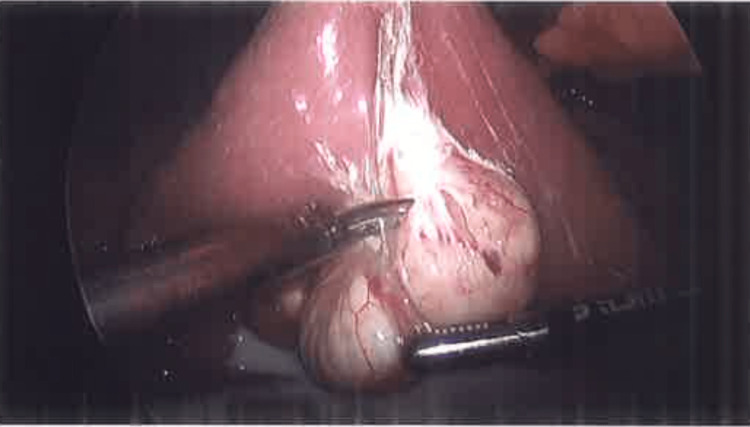
Image taken by laparoscopic camera showing the excision of the cyst from the jejunum.

In our case, the patient presented with abdominal pain in right lower quadrant, constipation, and nausea. In abdominal CT, a cystic lesion with fluid was identified in the patient. During the laparoscopic procedure, the mesenteric cyst in our patient was identified as wrapped around the jejunum. It was a multiloculated cyst and covered approximately 8 cm of the jejunum and around 70% of the circumference. We initially thought about resecting that part of the jejunum. However, the cyst came off fairly easily and was resected and sent to pathology (Figure 3). The pathology report confirmed connective tissue with cystic change consistent with mesenteric cysts. The mesenteric cyst fluid contained amorphous and fibrinoid material. It consisted of cystic fatty tissue in milky liquid. The tissue that was sent to pathology measured 2.5 x 1.8 x 0.6 cm.

## Conclusions

Before deciding on the optimal treatment plan for a patient presenting with a mesenteric cyst, a thorough evaluation should be conducted based on the patient's clinical presentation, imaging/laboratory studies, past medical history, and past surgical history to reach a provisional diagnosis. In our case, the patient’s mesenteric cyst was discovered in the jejunum, and the multilocular cyst contained chylous fluid, which was confirmed with postoperative pathology. For patients with clinical symptoms such as abdominal pain, nausea, and constipation, the definitive treatment is complete surgical removal to avoid recurrence and possible malignant transformation. Also, total cystectomy could be considered as a potential treatment in certain cases, such as our patient, to prevent potential secondary complications such as volvulus, spillage of infective fluid, herniation of bowel into an abdominal defect, or obstruction.
